# Molecular epidemiology and genetic characteristics of influenza viruses in a local pediatric population of eastern China, 2024

**DOI:** 10.3389/fmicb.2026.1799181

**Published:** 2026-04-16

**Authors:** Xiaoqing Sun, Xiang Li, Haiyan Chen, Tianyuan Lu, Zhaoqi Fang, Danyang Chen, Qiliang Cai, Jun Shen, Youhua Xie

**Affiliations:** 1 Key Laboratory of Medical Molecular Virology (MOE/NHC/CAMS), Shanghai Institute of Infectious Disease and Biosecurity, Shanghai Frontiers Science Center of Pathogenic Microorganisms and Infection, School of Basic Medical Sciences, Shanghai Medical College, Fudan University, Shanghai, China; 2 Pediatric Department, Children's Hospital of Fudan University at Qidong, Nantong, China; 3 Infectious Disease Department, Children's Hospital of Fudan University, National Children's Medical Center, Shanghai, China

**Keywords:** East China, influenza virus, molecular epidemiology, pediatric population, whole-genome sequencing

## Abstract

**Introduction:**

The persistent circulation of influenza viruses following the COVID-19 pandemic remains a major public health concern, yet local genomic epidemiology in pediatric populations is not well-defined.

**Methods:**

In 2024, we collected 490 influenza antigen-positive specimens from a pediatric hospital in Qidong, eastern China, and performed influenza genome enrichment-based whole-genome sequencing, yielding 425 genomes (86.73%) with at least hemagglutinin (HA) and neuraminidase (NA) segments.

**Results:**

A/H1N1 (45.41%), A/H3N2 (18.35%), and B/Victoria (36.24%) co-circulated, with two activity peaks in January and December. Phylogenetic analysis assigned these viruses to clades 6B.1A.5a.2a (A/H1N1), 3C.2a1b.2a.2a.3a.1 (A/H3N2), and V1A.3a.2 (B/Victoria). Positively selected sites in HA included lineage-specific residues such as position 210 in B/Victoria and positions 187 and 372 in A/H3N2. The oseltamivir resistance marker NA-H275Y was detected in 7/193 (3.65%) A/H1N1 strains, whereas no detected resistance to RNA polymerase inhibitors.

**Discussion:**

These findings highlight the co-circulation patterns, genetic diversity, and antiviral susceptibility profile of influenza viruses in a pediatric population in eastern China, supporting the importance of sustained genomic surveillance to guide local prevention strategies and clinical management.

## Introduction

Influenza viruses remain a global public health concern, with estimated annual infection rates of 5%−10% in the general population and 20%−30% among children, imposing a considerable burden on healthcare systems worldwide (Iuliano et al., 2018). The clinical symptoms of influenza infection, including fever, cough, sore throat, and generalized fatigue, are nonspecific and often clinically indistinguishable from other acute respiratory illnesses. Seasonal influenza epidemics contribute to approximately 290,000–650,000 respiratory-associated deaths annually worldwide, with mortality disproportionately concentrated in high-risk populations such as elders (≥65 years), young children, pregnant women, and those with chronic comorbidities (e.g., cardiovascular diseases, diabetes mellitus, and chronic respiratory conditions) (Russell et al., 2008). Therefore, it is of significance to strengthen influenza surveillance in high-risk populations, promote vaccination, and ensure early diagnosis and appropriate antiviral therapy (Tafalla et al., 2016).

Influenza viruses, belonging to the *Orthomyxoviridae* family, are enveloped viruses with a segmented, negative-sense single-stranded RNA viruses consisting of eight distinct RNA segments (Chauhan and Gordon, 2022). These segments encode eight structural proteins (PB2, PB1, PA, HA, NP, NA, M1, and M2) and two non-structural proteins (NS1 and NS2). Influenza viruses can be classified into four types (A, B, C, and D), of which types A and B are the primary agents responsible for seasonal influenza in humans (Chauhan and Gordon, 2022). Current epidemiological surveillance indicates that the dominant circulating strains are influenza A subtypes (H1N1pdm09 and H3N2) and the influenza B/Victoria lineage (Russell et al., 2008). Among the viral proteins, hemagglutinin (HA) and neuraminidase (NA) are key targets of the host immune response and antiviral drugs, respectively. Frequent mutations in these proteins drive antigenic drift and shift, which can result in vaccine failure and the emergence of novel strains (Javanian et al., 2021; Lowen, 2017). In the context of vaccine strain selection, monitoring antigenic variation in HA is of paramount importance. The H1N1pdm09 HA1 subunit contains five antigenic regions (Sa, Sb, Ca1, Ca2, and Cb), while the H3N2 HA1 subunit is characterized by five antigenic sites (A, B, C, D, and E) (Popova et al., 2012; Miron et al., 2021). In contrast, the B/Victoria lineage mainly involves four key antigenic determinants located in the 120-loop, 150-loop, 160-loop, and 190-helix regions (Suntronwong et al., 2021). Monitoring variations at these positions is essential for understanding influenza transmission dynamics and guiding the selection of optimal vaccine candidates (Zeng et al., 2024).

The clinical treatment of seasonal influenza primarily relies on antiviral agents targeting NA or RNA polymerase (Lampejo, 2020). Representative NA inhibitors (NAIs) include oseltamivir, zanamivir, and peramivir, among others. Global surveillance data indicate a low resistance rate (approximately 1–2%) among H1N1 strains to oseltamivir, the most widely prescribed NAI (Zhang et al., 2022; Renaud et al., 2011). RNA polymerase inhibitors, such as baloxavir marboxil and favipiravir, target the viral RNA-dependent RNA polymerase complex (comprising PA, PB1, and PB2 subunits), disrupting viral RNA replication (Hayden and Shindo, 2019). Reported resistance rates to baloxavir marboxil range from 2% to 10%, with higher frequencies observed in pediatric populations (Giacchello et al., 2021; Abed et al., 2021). Clinical evidence demonstrates that antiviral administration within 48 hours of symptom onset significantly reduces the risks of severe complications and mortality. Early intervention is particularly crucial for immunocompromised individuals and other high-risk groups.

The global implementation of whole-genome sequencing (WGS) for influenza surveillance has been propelled by the rapid development and adoption of high-throughput sequencing (HTS) technologies (Van Poelvoorde et al., 2021; Hu et al., 2021). Compared to traditional surveillance approaches focusing solely on HA gene analysis, influenza WGS enables comprehensive acquisition of all eight genomic segments in a single workflow (Galli et al., 2022). This approach enhances the resolution of spatiotemporal epidemiological dynamics, facilitates the detection of inter-subtype recombination events, identifies mutations across the entire genome, and refines genotype classification (Chon et al., 2023). In this study, we applied whole genome sequences (WGS) to influenza-positive specimens collected from a pediatric hospital in Qidong, a coastal region in eastern China, during 2024. Our analysis characterized subtype-specific prevalence, identified putative drug-resistant mutations, and assessed antigenic variation across circulating strains.

## Materials and methods

### Samples collection

In this study, influenza-positive samples were collected from a pediatric hospital in Qidong, a coastal region of eastern China, during the seasonal peaks in 2024, spanning from January to May and November–December. Nasopharyngeal swabs were obtained from patients presenting with influenza-like respiratory symptoms. Specimens were first screened using a SOFIA Influenza A and B Fluorescent Immunoassay (FIA) for rapid influenza antigen detection. Positive samples were immediately preserved in viral transport medium, flash-frozen at −80 °C, and subsequently subjected to high-throughput WGS.

### RNA isolation, PCR amplification, and whole-genome sequencing

Viral RNA was extracted from positive swabs using the MiniBEST Viral RNA/DNA Extraction Kit (TaKaRa) according to the manufacturer's instructions. Complementary DNA (cDNA) was synthesized with influenza A and B universal primers using the PrimeScript II 1st strand cDNA Synthesis Kit (TaKaRa). Whole-genome amplification was carried out with the MBTuni-12 (ACGCGTGATCAGCAAAAGCAGG) and MBTuni-13 (ACGCGTGATCAGTAGAAACAAGG) primers for influenza A, and 13 primers for influenza B as described previously (Zhou et al., 2014; Van den Hoecke et al., 2015). For both virus types, PCR was performed using NEBNext Ultra II Q5 Master Mix (NEB) with 5 μL of cDNA under the following cycling conditions: initial denaturation at 98 °C for 30 s, followed by 30 cycles of denaturation at 98 °C for 10 s, annealing at 57 °C for 10 s, and extension at 72 °C for 2 min. Amplified products were purified with VAHTS DNA Clean Beads (Vazyme) and quantified using a Qubit 4 fluorometer (Invitrogen) with the Qubit dsDNA BR Assay kit.

Sequencing libraries were prepared from the purified RT-PCR products using the Nextera XT DNA Sample Preparation Kit (Illumina) following the manufacturer's instructions. The amplified libraries were purified with 0.8 × VAHTS DNA Clean Beads, quantified using the Qubit dsDNA HS Assay Kit (Invitrogen), and assessed for fragment size on an Agilent 2100 Bioanalyzer System with the Agilent High Sensitivity DNA Kit (Agilent). All qualified libraries were pooled and sequenced on an Illumina NextSeq 2000 platform using v3 chemistry, generating 2 × 150 bp paired-end reads according to the standard protocol.

### Generation of consensus genome sequences

All raw sequencing reads were filtered with FastQC v0.11.9. Adapters and low-quality bases were then trimmed using fastp 0.23.4, retaining only reads with a Phred quality score ≥20. The filtered reads were re-evaluated with FastQC v0.11.9 to confirm data integrity prior to downstream analysis. Subsequently, the processed reads were assembled and screened for genomic variations using the Influenza Realtime Application (IRMA v1.2.0) pipeline (https://github.com/CDCgov/irma), a high-throughput bioinformatics tool designed for reference-based assembly of segmented RNA viruses such as influenza, Ebola, and coronaviruses (Shepard et al., 2016). The IRMA used BLAST, Samtools and minimap2 for the major search, alignment construction and assembly process, and default parameters were used.

### Phylogenetic analysis

Phylogenetic analysis was performed using all assembled full-length influenza virus sequences, combined with reference sequences obtained from the NCBI and GISAID databases. Only complete influenza genomes (A/H1N1, A/H3N2, and B/Victoria) containing all eight gene segments were downloaded. Sequences collected between 2014 and 2024 were retained. Duplicated sequences present in both GISAID and NCBI were removed. A subset of 5,000 reference sequences was sampled with SMOT (https://github.com/flu-crew/smot) from preliminary tree constructed using whole HA sequences to reduce overrepresentation from highly sampled regions or time periods while preserving major lineage diversity. We then built another phylogenetic tree with IQ-TREE v2.3.6 by using 5,000 reference sequences generated, and outlier sequences were removed with TempEst v1.5.3. Sequences without detailed collection date and with large root-to-tip regression distance were removed. A further refined dataset with 800 sequences was selected to build the final time-resolved phylogenetic tree. The selected dataset has a good topology with low quality sequences removed and relative balanced diversity across collection dates and geographic diversity.

This final curated reference dataset, together with corresponding influenza vaccine strains and the 2024 circulating strains from Qidong, was used to construct the phylogenetic tree with BEAST v1.10.4. The analysis implemented using a HKY + Gamma model, an uncorrelated lognormal relaxed clock model, and a Gaussian Markov random field (GMRF) Bayesian Skyride coalescent prior. Each Markov chain Monte Carlo (MCMC) run was performed for 100 million states, with sampling every 10,000 states and a 10% burn-in discarded after evaluation with Tracer v1.7.2. The maximum clade credibility (MCC) trees were summarized by TreeAnnotator within the BEAST package and visualized with R package ggtree.

### Antigenicity prediction and antigenic mapping using PLANT

To infer the antigenic relationships of locally circulating influenza A/H3N2 viruses, we applied PLANT (Protein Language Model for Antigenic cartography), which projects influenza A/H3N2 HA protein sequences onto a pre-constructed antigenic map. HA1 amino-acid sequences were extracted from our assembled genomes and used as model inputs (Ito et al., 2025). Antigenic coordinates were predicted using the pretrained “full model”, and we used the recommended PLANT_fixed variant (v1.1) for inference (https://github.com/TheSatoLab/PLANT). Local sequences were embedded and overlaid onto the reference antigenic map following the authors' provided inference workflow with Google Colab notebook, without additional model training. Antigenic proximity among strains (and between local strains and selected reference/vaccine strains) was summarized using distances derived from the predicted map coordinates. PLANT performs inference using HA1 protein sequences (reference and target strains) and experimental metadata (e.g., clade) with a protein language model (ESM-2) to embed HA1 sequences and predict 3D coordinates on an antigenic map. Antigenic map was plotted with predicted antigenic distance.

### Determination of selection pressure

Positive selection analysis was conducted based on the ratio of non-synonymous to synonymous substitutions (dN/dS). Putative sites under selection were statistically evaluated using the Mixed Effects Model of Evolution (MEME) and the Fast Unconstrained Bayesian AppRoximation (FUBAR) methods, implemented in the HYPHY software suite via the Datamonkey web server (https://www.datamonkey.org/). For the MEME method, a site was considered under significant positive selection if the *p*-value was < 0.1. For the FUBAR method, a posterior probability threshold > 0.9 was applied to identify sites subject to pervasive positive or negative selection.

### Identification of sequence mutations

Mutation analysis for all sequences was conducted using the web-based platform of Nextstrain (https://nextstrain.org/). The specific procedure involved accessing the Nextclade module, submitting our curated sequence set, and selecting the most phylogenetically closely related sequences as the reference strains for comparative mutation profiling.

### Data availability

The sequences reported in this study have been deposited in GISAID databases, which can be accessed through the following link (ID: EPI_SET_260311cu, https://doi.org/10.55876/gis8.260311cu) (Supplementary Figure 1).

### Ethics statement

All procedures were conducted in accordance with the ethical standards of the Medical Ethics Council of Qidong Affiliated Hospital, Children's Hospital of Fudan University (Number QDFY2024001). Informed consent was obtained from all participants.

## Results

### Study population

During seasonal peaks in 2024, a total of 490 influenza antigen-positive samples were collected from a pediatric hospital in Qidong, a coastal region in eastern China, as identified by rapid influenza antigen detection. This cohort exhibited a balanced gender distribution, with females accounting for 53.1% of cases. Most patients were aged 3–5 years (26.35%, *n* = 112) or 6–17 years (55.06%, *n* = 234), while smaller proportions were aged 0–2 years (7.6%) or over 18 years (5.1%). Therefore, the study sample predominantly consisted of pediatric cases (94.9%), with a small proportion of adult cases (5.1%). All enrolled patients presented with influenza-like illness, characterized by fever, cough, and rhinorrhea. Following clinical evaluation, nasopharyngeal swabs specimens were collected for rapid antigen testing. Samples with positive results were subsequently subjected to influenza genome enrichment-based WGS (Supplementary Table 1).

### Epidemiological profile of influenza viruses

**Figure 1:**
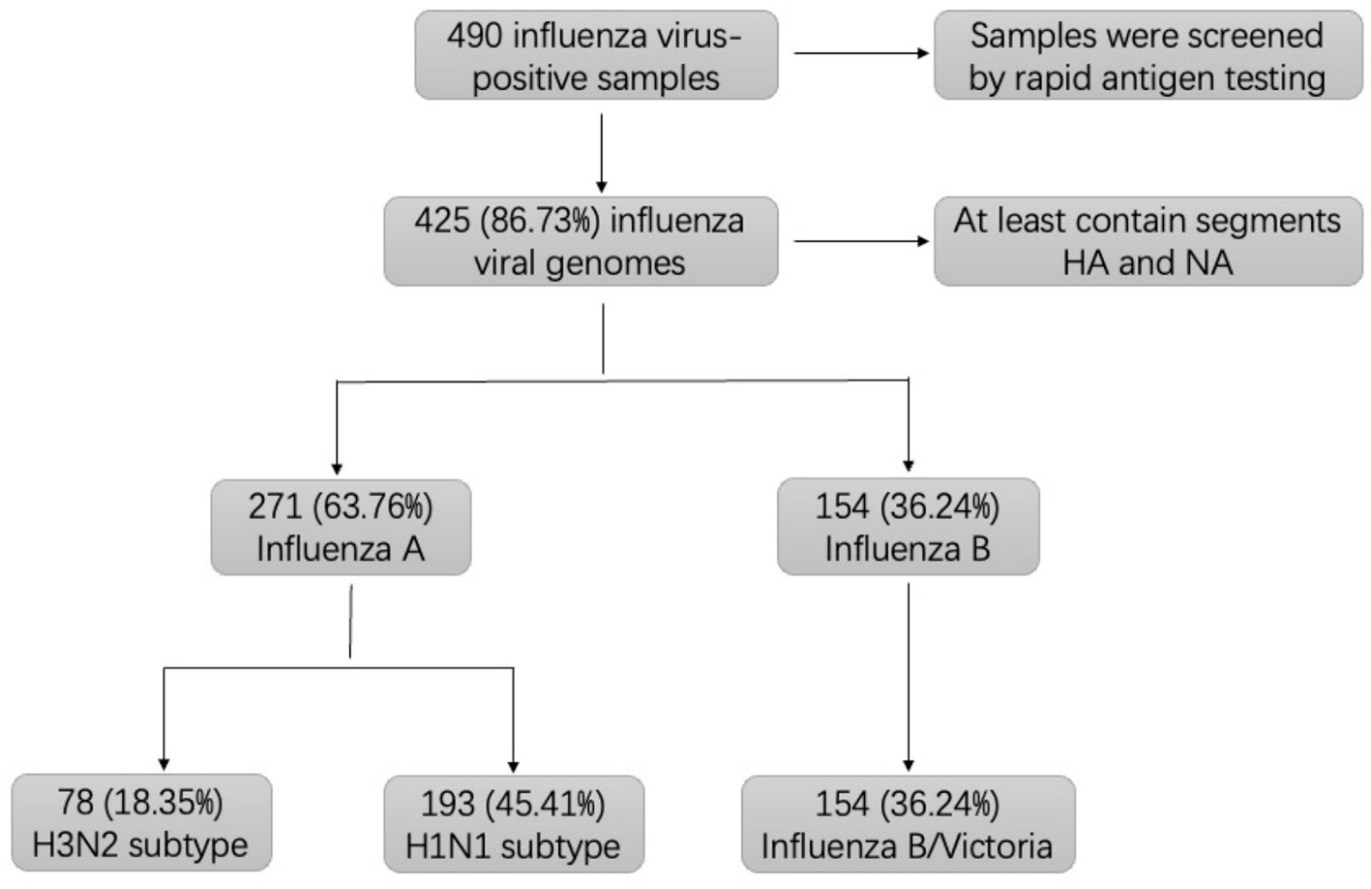
Flowchart of sample collection and influenza virus characterization. A total of 490 influenza antigen-positive samples were collected from a pediatric hospital in Qidong, a coastal region of eastern China. Percentages of influenza virus genotypes and subtypes were calculated related to the total number of successfully sequenced influenza cases following whole-genome sequencing and analysis.

**Figure 2:**
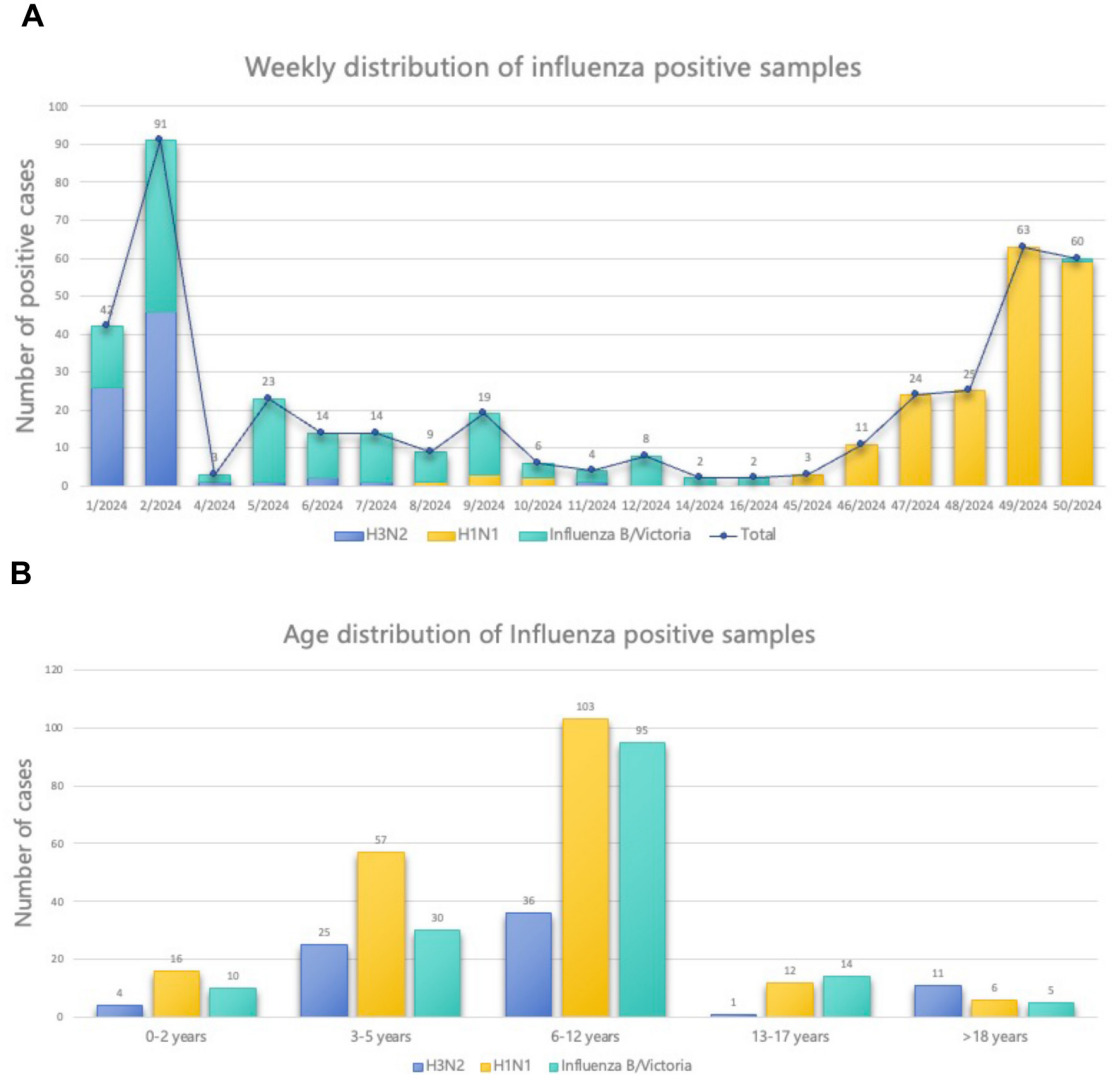
Weekly and age distribution of influenza virus cases. **(A)**. Weekly distribution of cases by influenza virus type in 2024. Numbers above the bars indicate the weekly count of influenza positive samples that were successfully sequenced. **(B)**. Age distribution of influenza virus cases for different genotypes in 2024.

Of the 490 samples, WGS successfully recovered 425 (86.73%) influenza virus genomes containing at least the HA and NA segments (Supplementary Figure 1). Genetic characterization identified 271 type A influenza viruses (63.76%), with A/H1N1 (45.41%) and A/H3N2 (18.35%) as the predominant subtypes. The remaining 154 sequenced genomes (36.24%) were all influenza B viruses, belonging to B/Victoria lineage (Figure 1). During the early phase of the winter-spring period in 2024 (weeks 1–12), A/H3N2 and B/Victoria were the dominant circulating strains (Figure 2A). As ambient temperatures increased, the prevalence of A/H3N2 declined, whereas B/Victoria continued to circulate. From week 45 onward, A/H1N1 emerged as the predominant subtype, with only sporadic detections of B/Victoria. Annual epidemiologic analysis revealed two distinct peaks of influenza activity, occurring in January (37.9%) and December 2024 (34%) (Figure 2A). As to age-distribution, most of influenza cases were 6–12 years old, followed by age 3–5 years (Figure 2B).

### Evolutionary and genetic characterization of influenza A/H1N1 strains

**Figure 3:**
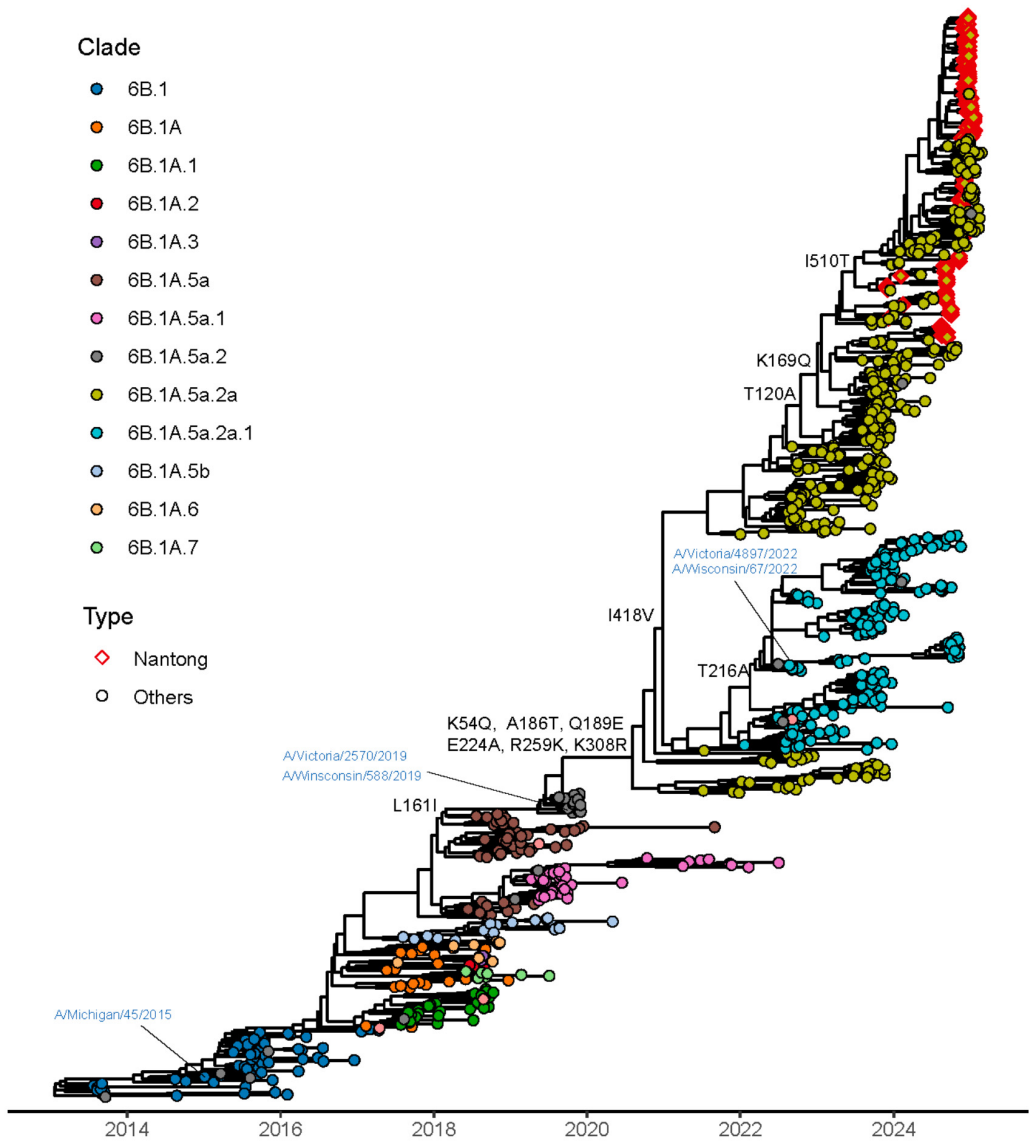
Phylogenetic analysis of the HA gene of influenza A(H1N1)pdm09. The maximum clade credibility tree was reconstructed using sequences from the Qidong region, globally representative reference strains, and vaccine strains. Phylogenetic inference was performed using BEAST v1.10.4, and the tree was visualized with the ggtree package in R. Major genetic clades are color-coded. Strains circulating in the Qidong region are highlighted with red diamonds.

**Figure 4:**
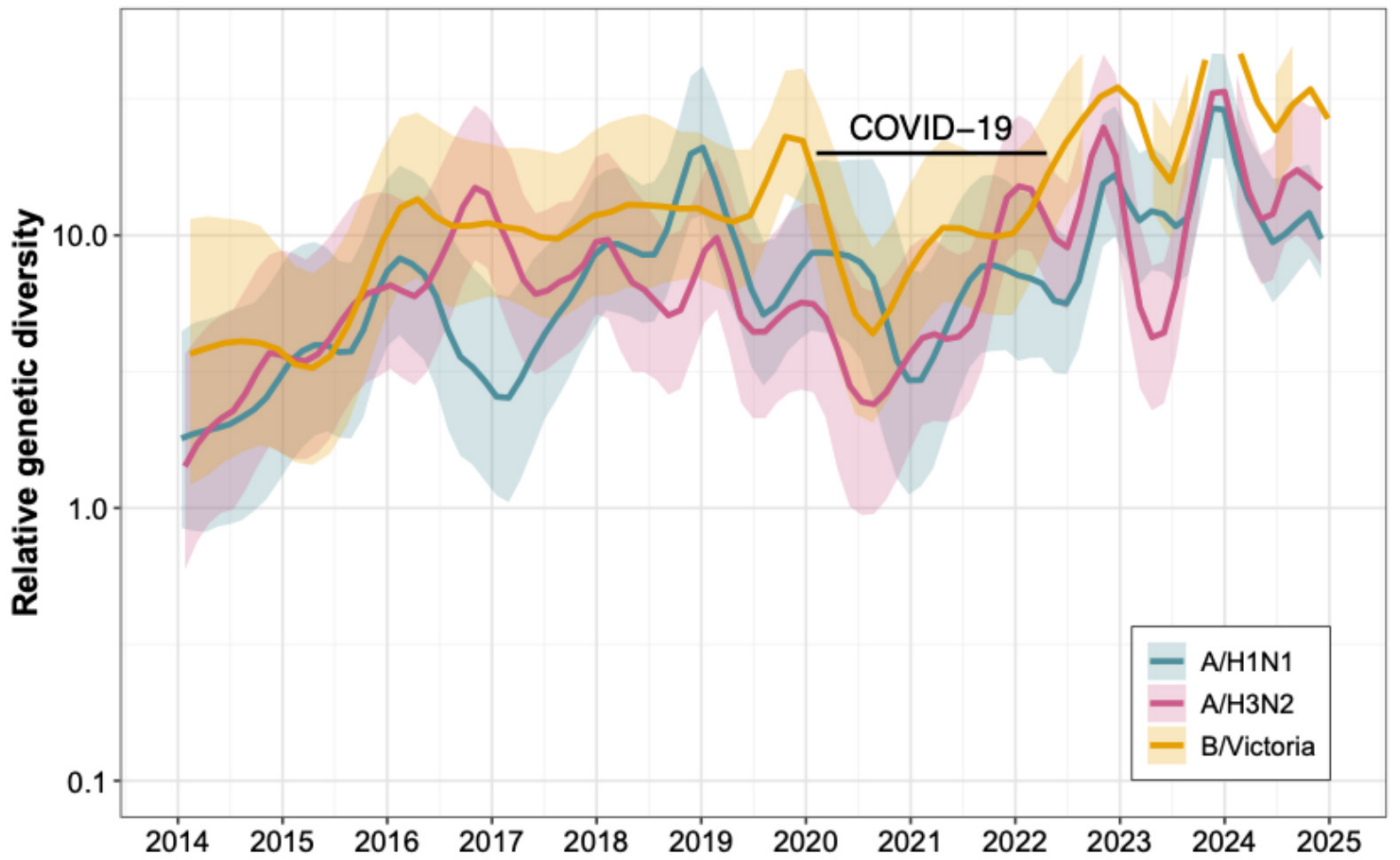
Gaussian Markov Random Field (GMRF) Bayesian Skyride analysis of the HA gene of influenza A (H1N1) pdm09, A(H3N2), and B/Victoria. The solid line representing the posterior mean effective population size, and the shaded area denotes the 95% highest posterior density (HPD) intervals.

Phylogenetic analysis was performed on the 193 A/H1N1 strains. To investigate the evolutionary dynamics beyond the HA gene, we also analyzed the NA and PA genes (Supplementary Figure 2). Comparing these trees with the HA phylogeny allows for the detection of segment reassortment and provides a more comprehensive view of the viral genomic evolution. Representative global reference sequences from the past decade were retrieved from the NCBI and GISAID databases and included together with Qidong sequences in the phylogenetic reconstruction to place the local strains within the broader global evolutionary context (Supplementary Table 2). All A/H1N1 strains circulating in Qidong, eastern China, in 2024 belonged to clade 6B.1A.5a.2a and could be further subdivided into two subclades: C1.9 and C1.9.3 (Figure 3). A detailed phylogenetic tree is provided in Supplementary Figure 3. The time to the most recent common ancestor (tMRCA) for strains from Qidong was estimated to be near May 2023. The phylogenetic tree showed that strains from Qidong clustered closely with viruses from Europe, North America, South America, and Asia, indicating that the local epidemic strains were embedded within contemporaneously circulating global lineages rather than forming a geographically restricted cluster. In addition, a Gaussian Markov Random Field (GMRF) Bayesian Skyride analysis of the HA gene indicated modest fluctuations in effective population size over the past decade, with alternating periods of slight expansion and contraction (Figure 4). It should be noted that the highest posterior density (HPD) intervals associated with the effective population size estimates are relatively wide, indicating considerable uncertainty in the inferred trajectories.

Selective pressure analysis on the H1N1 HA gene yielded method-dependent results. The Mixed Effects Model of Evolution (MEME) detected no sites under statistically supported episodic positive selection, whereas the Fast Unconstrained Bayesian AppRoximation (FUBAR) method identified one codon (position 207) under positive selection and 14 codons under negative selection (Supplementary Table 3).

Comparative analysis of HA, NA, and PA sequences from Qidong identified several mutations in the HA gene. The substitution D222N (1.55%, 3/193) found among Qidong strains and has been reported to affect receptor-binding properties of influenza viruses (Kong et al., 2014; Kolosova et al., 2023; Houng et al., 2012). In addition, S190R has been implicated in altered pathogenicity in prior studies (Chen Y. et al., 2018). Beyond its role in antigenicity, the NA protein is the primary target of antiviral development. In NA, 7 of 193 strains (3.65%) harbored the well-characterized oseltamivir resistance substitution H275Y, and additional substitutions previously associated with reduced NA inhibitor susceptibility—including S247N, D199N, S200N, S339L, and G282R—were also observed (Xu et al., 2024). Analysis of the PA sequences revealed that Qidong A/H1N1 strains retained substitutions such as V100I, P224S, N321K, I330V, and R362K, which have been reported in association with increased virulence or polymerase activity in influenza viruses (Nogales et al., 2018). No established resistance-associated substitutions to RNA polymerase inhibitors (e.g., baloxavir marboxil) (Omoto et al., 2018) were detected (Supplementary Table 4).

### Evolutionary and genetic characterization of influenza A/H3N2 viruses

**Figure 5:**
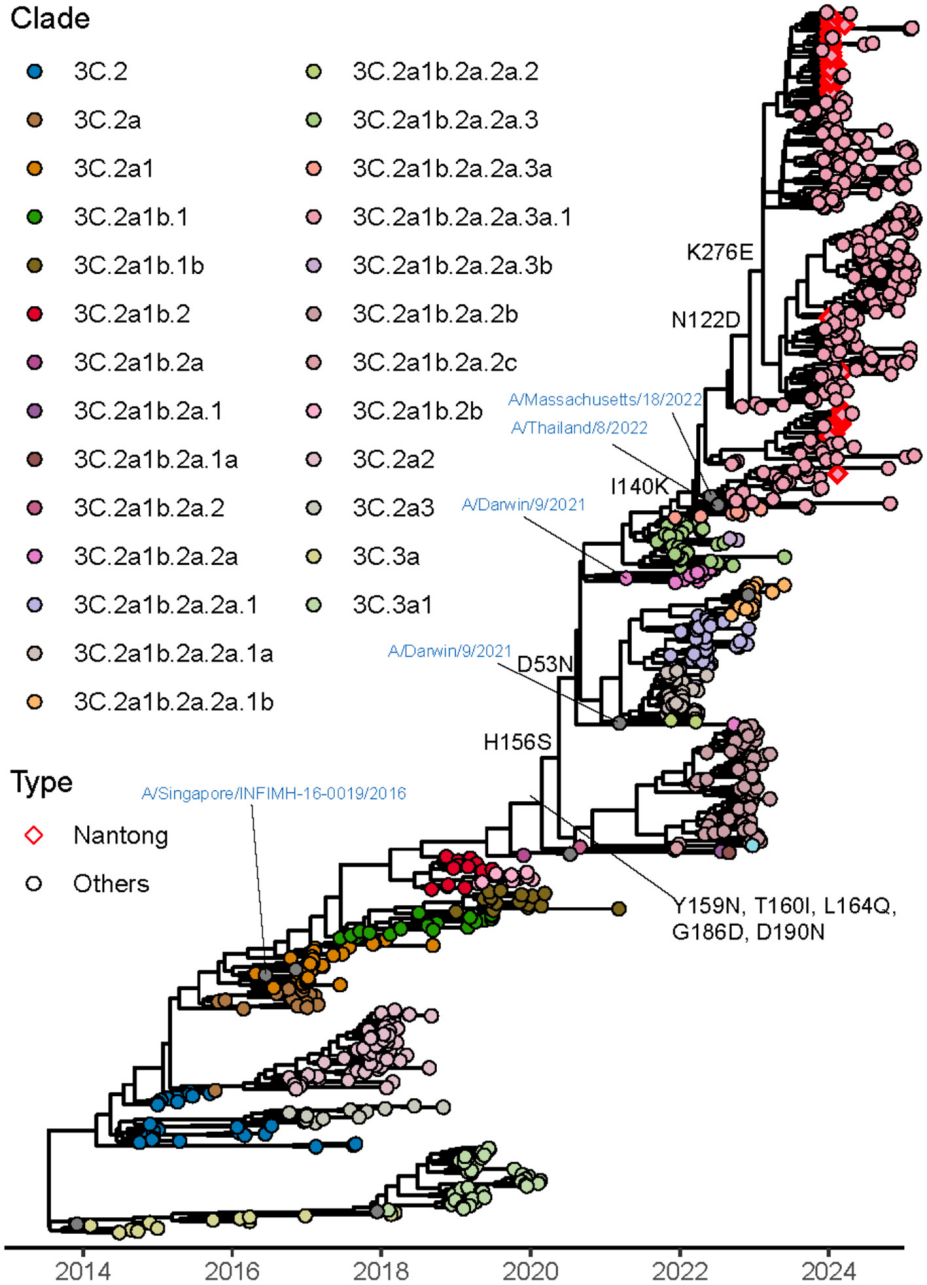
Phylogenetic analysis of the HA gene of influenza A(H3N2). The maximum clade credibility tree was reconstructed using sequences from the Qidong region, globally representative reference strains, and vaccine strains. Phylogenetic inference was performed using BEAST v1.10.4, and the tree was visualized with the ggtree package in R. Major genetic clades are color-coded. Strains circulating in the Qidong region are highlighted with red diamonds.

Phylogenetic analysis was performed on the 78 A/H3N2 strains obtained in this study using their HA, NA, and PA gene segments. To capture global evolutionary trends, H3N2 reference sequences from the past decade were retrieved from the NCBI and GISAID databases and incorporated into the phylogenetic reconstruction (Figure 5). All A/H3N2 strains were classified into clade 3C.2a1b.2a.2a.3a.1, which could be further subdivided into four subclades: J.1, J.2, J2.1, and J.3 (see Supplementary Figure 4 for a detailed phylogenetic tree). The time to the most recent common ancestor (tMRCA) for strains from Qidong was estimated to be near January 2023. The phylogenetic tree indicated that these strains clustered on the same branch as contemporary strains from Europe, North America, South America, and Asia. In addition, GMRF Bayesian Skyride analysis illustrated the temporal dynamics of the effective population size of H3N2 viruses over the last 10 years (Figure 4).

**Figure 6:**
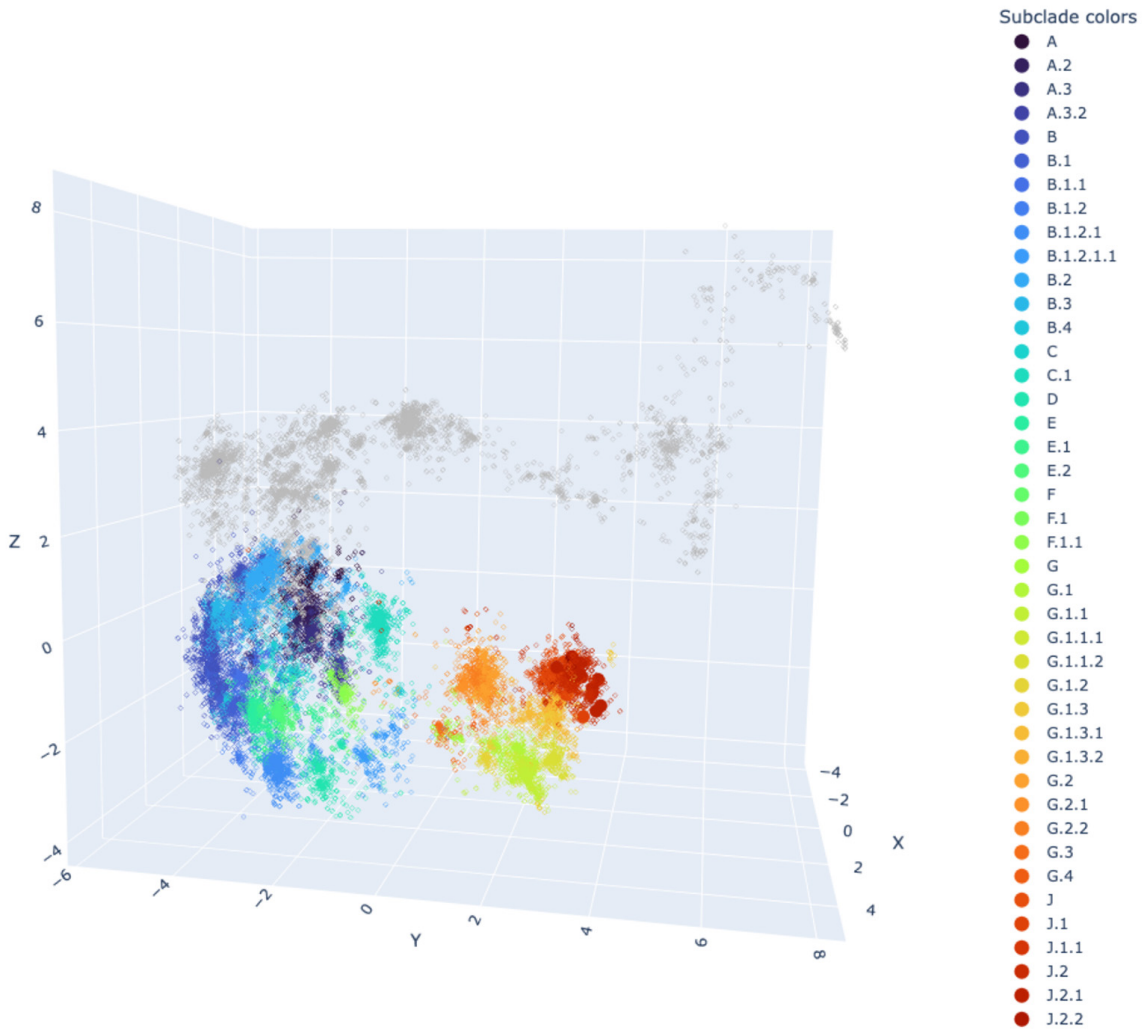
Antigenic map of influenza A/H3N2 strains based on the HA1 region, constructed using the Protein Language Model for Antigenic cartography (PLANT). HA1 amino-acid sequences were extracted from our assembled genomes and used as model inputs. Antigenic coordinates were predicted using the pretrained “full model”, and the recommended PLANT_fixed variant (v1.1) were used for inference.

We also employed PLANT (Protein Language Model for Antigenic cartography) to construct an antigenic map of A/H3N2 based on the HA1 region (Figure 6). Antigenic cartography can help identify antigenic differences in HA between newly circulating strains and previously prevalent strains, thereby enabling faster detection and identification of variants with potential immune escape. The map showed that the A/H3N2 strains circulating in the Qidong area (J.1, J.2, J.2.1, J.3) are relatively clustered within a single grid (Figure 6). This clustering pattern suggests relatively limited antigenic divergence among the circulating strains and indicates that they remain antigenically close to the vaccine reference within the modeled antigenic landscape. However, as the PLANT framework provides predictive antigenic estimates based on sequence information, serological measurements are required for validation.

Analysis of selective pressure on the H3N2 HA gene produced method-dependent outcomes. The Mixed Effects Model of Evolution (MEME) identified two sites under positive selection that approached statistical significance, specifically at positions 372 (*p* = 0.062) and 187 (*p* = 0.099). In contrast, the Fast Unconstrained Bayesian Approximation (FUBAR) method detected seven sites under negative selection. (Supplementary Table 3).

Comparative analysis of the HA, NA, and PA genes was performed for the A/H3N2 strains. We identified several HA mutations located within five major antigenic regions of H3N2, including sites in region A (N122D, S144N), region B (I160T, I192F), region C (E50K, G53N, K276E), region D (P227S), and region E (G62E, N63K, N94S). These mutations, individually or in combination, may contribute to antigenic differences in the H3N2 strains.

Analysis of the NA sequences did not reveal any known mutations conferring strong resistance to antiviral drugs. However, the mutations K267N and H264N were found to introduce a new glycosylation site, which may influence viral antigenicity. Other mutations, such as S331G and L134I, were identified and may be associated with moderate resistance to NA inhibitors. No mutations linked to resistance against RNA polymerase inhibitors were detected in the PA segments sequenced. All HA, NA and PA mutations identified are summarized in Supplementary Table 4.

### Evolutionary and genetic characterization of influenza B/Victoria viruses

**Figure 7:**
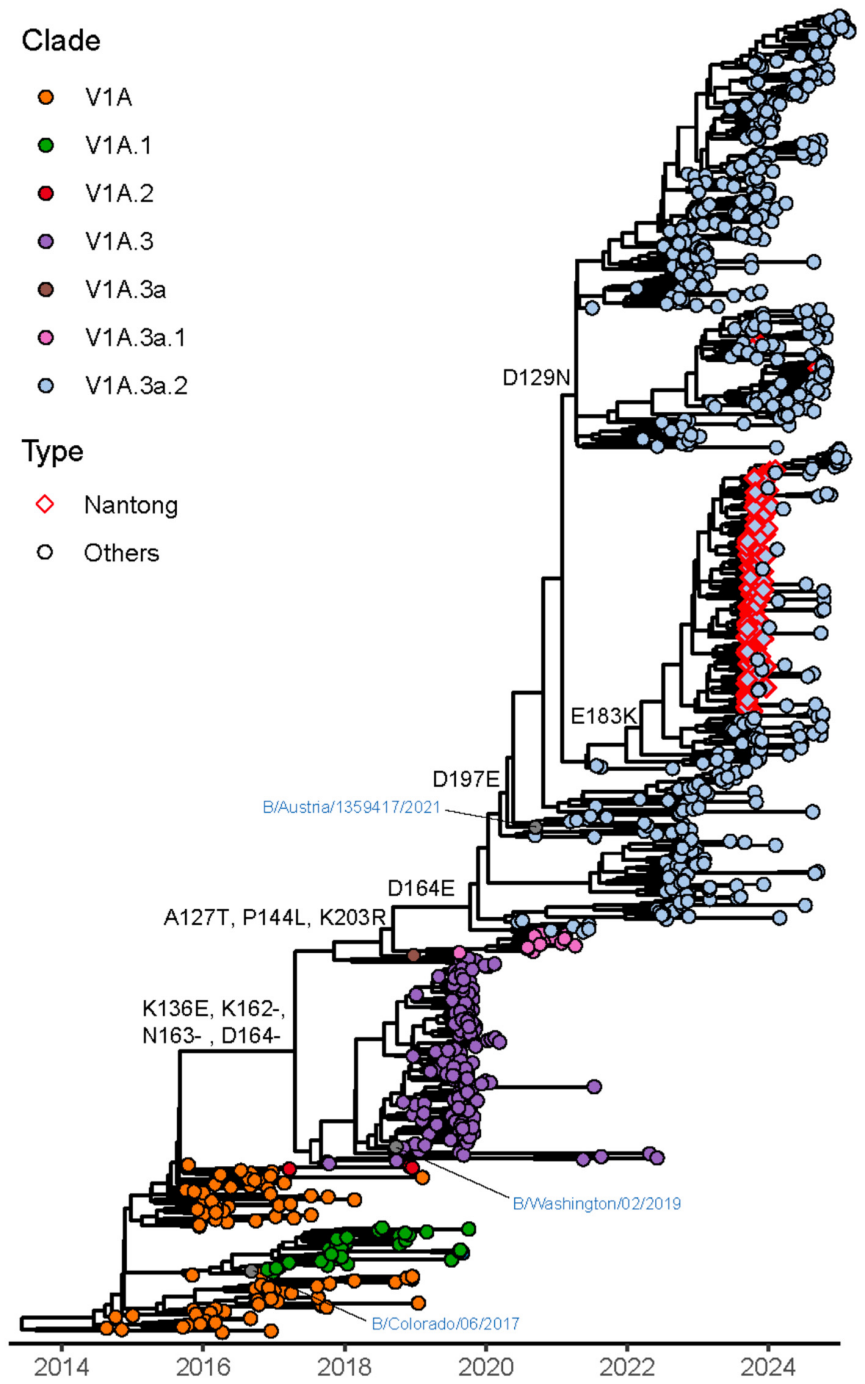
Phylogenetic analyses of the HA gene of influenza B virus. The maximum clade credibility tree was reconstructed using sequences from the Qidong region, globally representative reference strains, and vaccine strains. Phylogenetic inference was performed using BEAST v1.10.4, and the tree was visualized with the ggtree package in R. Major genetic clades are color-coded. Strains circulating in the Qidong region are highlighted with red diamonds.

The same methodology was applied to the sequenced influenza B virus strains. Phylogenetic analysis based on the HA, NA and PA segments was performed (Figure 7). All influenza B viruses circulating in the Qidong region in 2024 were identified as belonging to the Victoria lineage, specifically falling within the V1A.3a.2 clade, which was further divided into C5.6 and C5.7 subclades (see Supplementary Figure 5 for a detailed phylogenetic tree). To provide a comprehensive contextual comparison, we also selected representative epidemic strains from the past decade from NCBI and GISAID as references (Supplementary Table 2). No strains of the B/Yamagata lineage were detected. The time to the most recent common ancestor (tMRCA) for strains from Qidong was estimated to be near January 2023. The GMRF Bayesian Skyride analysis for influenza B viruses also revealed a progressive increase in the effective population size throughout the past decade (Figure 4).

Analysis of selective pressure on the B/Victoria HA gene revealed method-specific findings. The Mixed Effects Model of Evolution (MEME) identified one site (position 210, *p* = 0.004) under significant positive selection. In comparison, the Fast Unconstrained Bayesian AppRoximation (FUBAR) method detected two sites under positive selection and 27 sites under negative selection (Supplementary Table 3). The identification of these positive selection sites varies across different methods, further validation is thus required to confirm them.

Further comparative analysis of the HA, NA, and PA genes of the B/Vicoria strains revealed that the 2024 prevalent strains retained the three-amino-acid deletion at positions 162–164 in the HA gene. Additionally, a series of mutations were also identified, several of which are located within known antigenic regions of HA, including the 120-loop (H116N, I117V, H122Y, A127T, E128G, N129D, K136E), 150-loop (P144L, I146V, N150K), and 190-helix (N197E, E198R, A199T, A202V). Mutations were also identified in the NA gene. Among these, the substitutions S58P and T286I resulted in the loss of a glycosylation site, which may influence the antigenicity of NA. Other mutations, such as I115V, S295R, D320G, G247D, E358K, I361T, and D384G, may be associated with reduced susceptibility to NA inhibitors (Xu et al., 2024; Alizadeh et al., 2025; Tang et al., 2021). Analysis of the PA sequences from all strains did not reveal any mutations known to confer resistance to RNA polymerase inhibitors (Supplementary Table 4).

## Discussion

The Qidong region, located in Jiangsu province of eastern China, lies at the confluence of the Yangtze River, the Yellow Sea, and the East China Sea—a key node within the East Asian-Australasian Flyway used by millions of migratory birds. Given that migratory birds serve as natural reservoirs for influenza viruses and facilitate viral reassortment, this ecological setting provides a unique interface for zoonotic virus introduction into humans. We therefore selected Qidong as a surveillance site to monitor the epidemiological and genetic characteristics of seasonal influenza in the local population, aiming to enable early detection of viruses with potential public health implications (Bi et al., 2024).

In this study, we implemented an influenza high-throughput sequencing approach to characterize influenza-positive clinical samples collected during 2024, with a focus on the pediatric population. Our phylogenetic analysis of HA, NA and PA segments revealed that all characterized influenza strains clustered within established clades: A/H1N1 strains belonged to the 6B.1A.5a.2a clade, B/Victoria viruses to the V1A.3a.2 subclade, and A/H3N2 isolates to the 3C.2A clade. Notably, these circulating strains shared evolutionary branches with the 2024 vaccine reference strains [A/Wisconsin/67/2022 (H1N1), B/Austria/1359417/2021 (Victoria), and A/Darwin/9/2021 (H3N2)], suggesting an absence of major antigenic drift events leading to novel clade emergence in the Qidong region. However, while this genetic similarity implies potential antigenic relatedness, vaccine effectiveness ultimately requires confirmation through serological assays such as hemagglutination inhibition or neutralization tests. Continuous intra-clade evolution was observed, highlighting the necessity to monitor incremental antigenic changes that may precede vaccine mismatch.

As the primary target of host immune responses, HA is directly associated with antigenic evolution (Webster et al., 1982). Mutations within major antigenic epitopes of the globular head region can alter protein surface structure, enabling immune escape and driving antigenic drift (Essere et al., 2013). Although our analysis indicated no major antigenic changes at the clade level, several amino acid substitutions (e.g., D222N, S190R) were identified at antigenically relevant positions. These mutations, while not currently conferring immune escape, may accumulate over time and eventually contribute to antigenic divergence. This highlights the importance of detailed HA profiling for tracking evolutionary dynamics and supporting timely identification of potential variants. However, it also should be noted that the antigenic positions inferred using the PLANT model represent computational predictions based on sequence features rather than experimentally determined antigenic measurements (e.g., hemagglutination inhibition assays). Therefore, these results should be interpreted cautiously and primarily provide a preliminary indication of potential antigenic relationships.

The NA protein, a critical glycoprotein on the surface of influenza viruses, facilitates viral release by cleaving sialic acid residues on host cell membranes and enhances viral penetration through respiratory mucosal barriers by removing sialic acids (Samji, 2009). Mutations in antigenic epitopes of NA may reduce antibody neutralization efficiency (Chen Y. Q. et al., 2018), while specific substitutions can confer resistance to NA inhibitors such as oseltamivir and peramivir (Xu et al., 2024; Zhang and Ross, 2024). In this study, 7 out of 193 H1N1 strains (3.3%) harbored the H275Y mutation, a well-documented resistance marker, consistent with its prevalence in seasonal H1N1. In the sequenced H3N2 strains, mutations S331G and L134I were detected and may be associated with moderate resistance to NA inhibitors, while none of the primary resistance mutations (R292K, E119V, and N294S) were identified. For B/Victoria strains, no known resistance-associated substitutions (e.g., D197E, I221T, H273Y) were observed (Xu et al., 2024; Alizadeh et al., 2025; Tang et al., 2021). Despite the absence of strong resistance mutations within this cohort, continuous surveillance of these sites remains critical, particularly for high-risk populations (e.g., children, the elderly, and immunocompromised patients).

Baloxavir marboxil targets the cap-dependent endonuclease activity of the PA subunit, inhibiting the “cap-snatching” process essential for viral transcription (Dias et al., 2009). Emergent resistance mutations, particularly I38T/M/F in PA, have been documented in laboratory and clinical settings (Ince et al., 2020). In our analysis, none of the sequenced influenza strains (*n* = 425) harbored PA-I38T/M/F mutations, indicating that these resistance markers have not yet penetrated the circulating influenza pool in Qidong. This baseline data provides important surveillance evidence that RNA polymerase inhibitors remain a viable therapeutic option in this region. Nevertheless, given that novel resistance pathways may continue to emerge, our findings underscore the importance of ongoing monitoring of PA mutations to inform public health strategies. Baloxavir was initially approved in China for patients aged ≥12 years, with pediatric licensure extended to ≥5 years in 2023 (Omoto et al., 2018). Given the predominance of 3–12-year-olds in our cohort (*n* = 398, 81.22%) and the limited exposure of Baloxavir in younger children, the absence of resistance mutations aligns with expected epidemiological patterns. However, active monitoring for PA-I38T/M/F mutations is of significant importance as pediatric usage of RNA polymerase inhibitors expands.

By establishing a genomic surveillance framework for the 2024 season in Qidong, this study addresses a gap in understanding influenza evolution within the pediatric population of eastern China. Our data provide insights into antigenic drift patterns, emergent mutations, and antiviral susceptibility profiles specific to this demographic. However, several limitations should be considered, including the single-center design, single-year timeframe, and lack of clinical outcome data to correlate genomic findings with disease severity. Future multi-center studies with extended surveillance periods and integrated clinical data are warranted to validate these observations and inform more targeted public health strategies.

## Data Availability

The datasets presented in this study can be found in online repositories. The names of the repository/repositories and accession number(s) can be found in the article/Supplementary material.

## Supplementary material

Supplementary Figure 1 All genome sequences and associated metadata.

Supplementary Figure 2 Phylogenic analysis of NA and PA gene sequences of influenza virus.

Supplementary Figure 3 Detailed phylogenetic tree analysis of the HA gene of influenza A (H1N1) pdm09.

Supplementary Figure 4 Detailed phylogenetic tree analysis of the HA gene of influenza A (H3N2).

Supplementary Figure 5 Detailed phylogenetic tree analysis of the HA gene of influenza B virus.

Supplementary Table 1 Metadata of the samples involved in this study.

Supplementary Table 2 All reference sequence information retrieved from database in this study.

Supplementary Table 3 Results of selective pressure analysis.

Supplementary Table 4 Results of mutation analysis of HA, NA and PA.
