# Supplementary material for: Molecular epidemiology and genetic characteristics of influenza viruses in a local pediatric population of eastern China, 2024
Source: Front Microbiol. 2026 Apr 16;17:1799181. doi: 10.3389/fmicb.2026.1799181 (PMC13128664; doi:10.3389/fmicb.2026.1799181)
Supplement: Supplementary Figure 1 — All genome sequences and associated metadata. [file Data_Sheet_1.pdf]

Supplementary Appendix

All genome sequences and associated metadata supporting the findings of this study can be accessed through the persistent digital object identifier https://doi.org/10.55876/gis8.260313pf

In addition to the minted DOI, GISAID also communicates the aggregation of GISAID accession numbers (EPI_ISL_IDs) through the corresponding EPI_SET_260313pf identifier to facilitate both, the acknowledgment of all data contributors and the direct retrieval of the underlying data from GISAID used in this study.

Influenza Virus Data Summary

GISAID Identifier	Digital Object Identifier	Number of individual viruses	Data Collection range	Number of countries/territories
EPI_SET_260313pf	https://doi.org/10.55876/gis8.260313pf	423	2024-01-03 to 2024-12-18	1
