# Supplementary figures and images for: Molecular epidemiology and genetic characteristics of influenza viruses in a local pediatric population of eastern China, 2024
Source: Front Microbiol. 2026 Apr 16;17:1799181. doi: 10.3389/fmicb.2026.1799181 (PMC13128664; doi:10.3389/fmicb.2026.1799181)

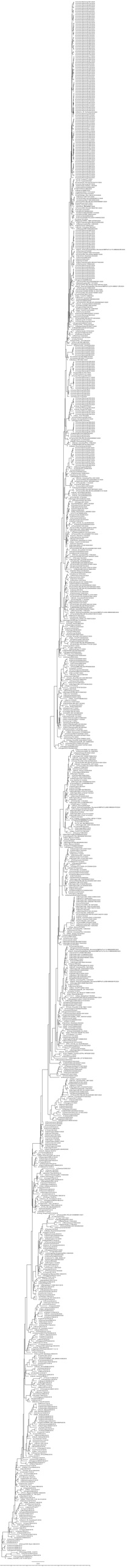

Supplement: Supplementary Figure 2 — Phylogenic analysis of NA and PA gene sequences of influenza virus. [file Data_Sheet_2.pdf]

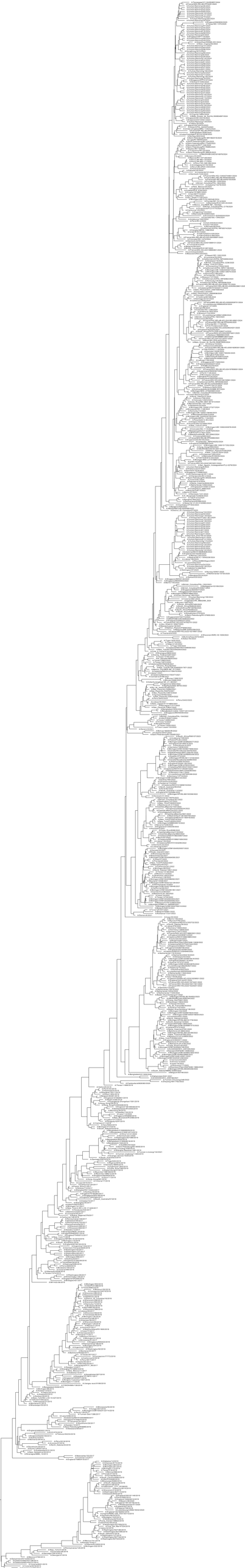

Supplement: Supplementary Figure 3 — Detailed phylogenetic tree analysis of the HA gene of influenza A (H1N1) pdm09. [file Data_Sheet_3.pdf]

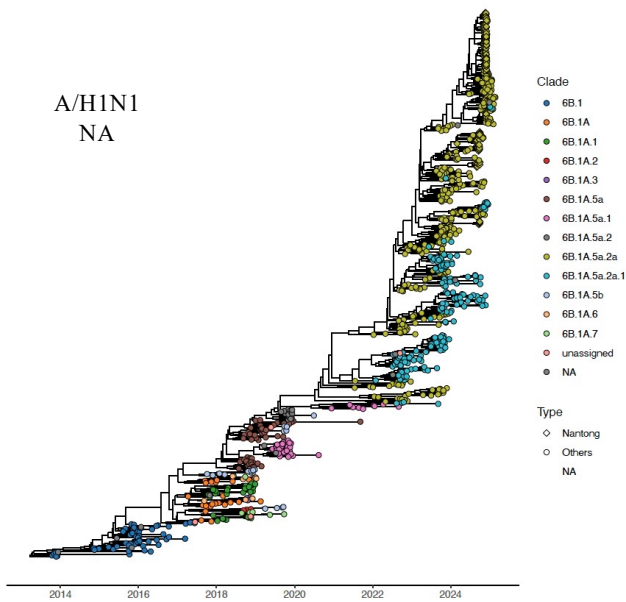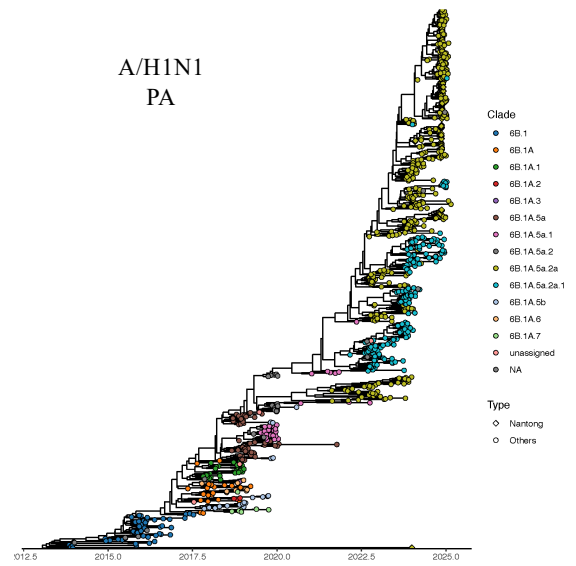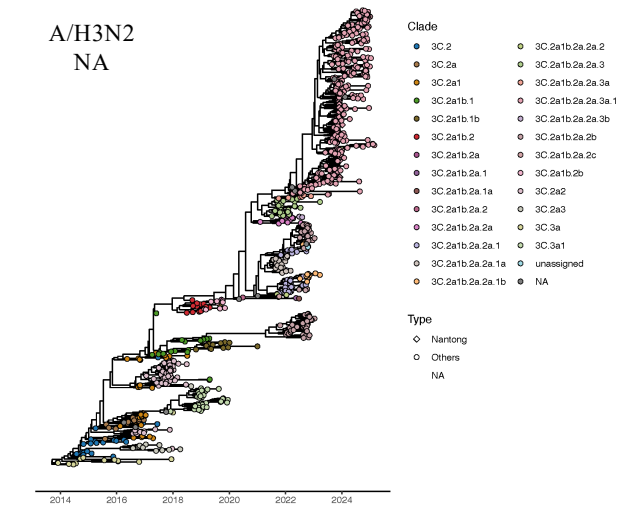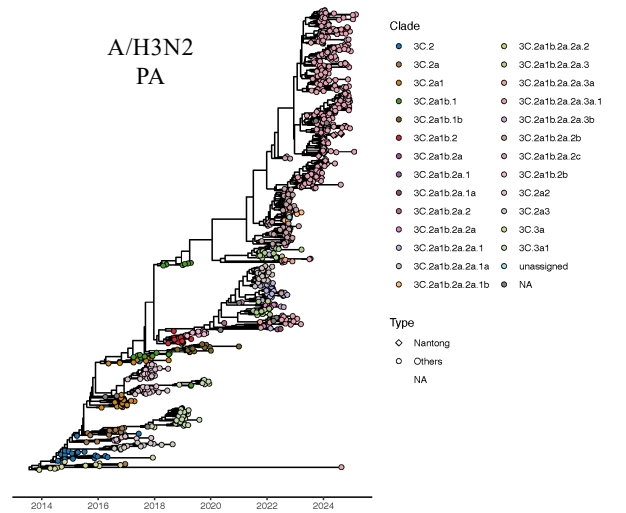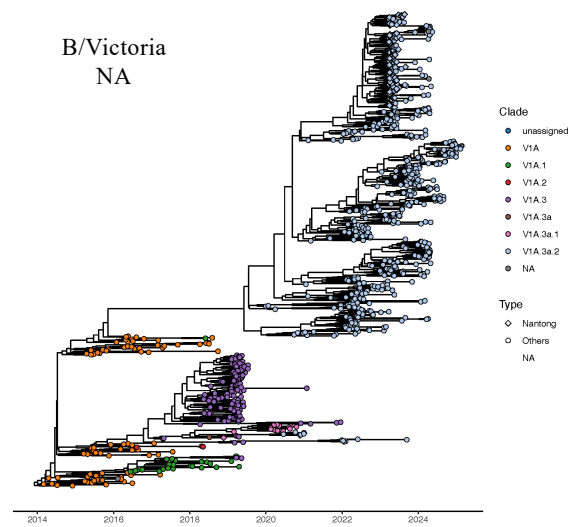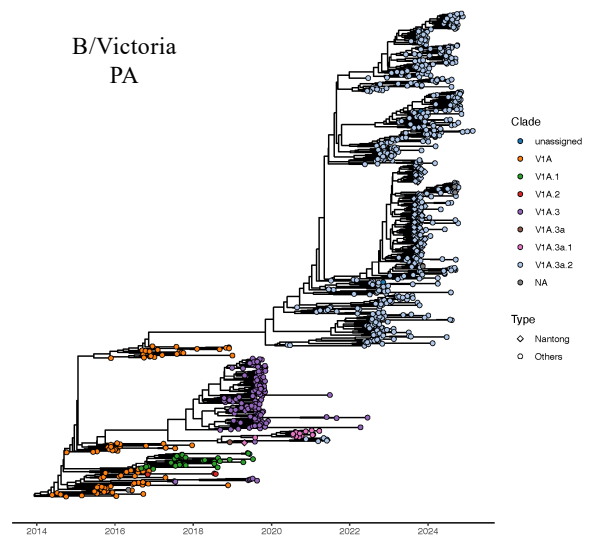

Supplement: Supplementary Figure 4 — Detailed phylogenetic tree analysis of the HA gene of influenza A (H3N2). [file Supplementary_file_1.pdf]

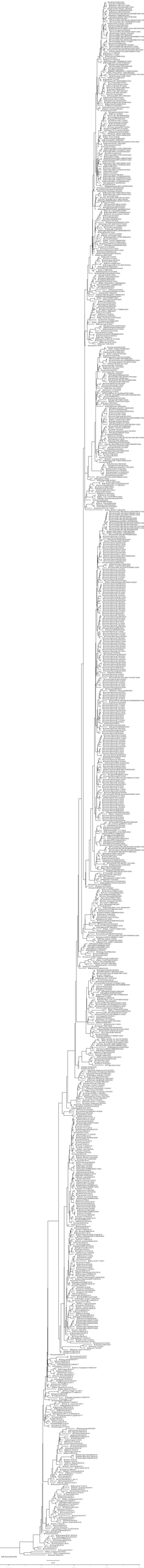

Supplement: Supplementary Figure 5 — Detailed phylogenetic tree analysis of the HA gene of influenza B virus. [file Data_Sheet_4.pdf]
